# Lifestyle Factors and Current Alcohol Consumption Among Japanese Adolescents During the COVID‐19 Pandemic: A Nationwide Cross‐Sectional Study

**DOI:** 10.1002/npr2.70089

**Published:** 2026-01-11

**Authors:** Masatake Nishiwaki, Hideyuki Kanda, Keita Yoshida, Takashi Hisamatsu, Aya Kinjo, Yuki Kuwabara, Hongja Kim, Aya Imamoto, Hisashi Yoshimoto, Teruna Ito, Hideaki Kasuga, Ruriko Minobe, Hitoshi Maesato, Maki Jike, Yuichiro Otsuka, Osamu Itani, Yoshitaka Kaneita, Susumu Higuchi, Yoneatsu Osaki

**Affiliations:** ^1^ Department of Public Health Okayama University Graduate School of Medicine, Dentistry, and Pharmaceutical Sciences Okayama Japan; ^2^ Division of Environmental and Preventive Medicine, Department of Social Medicine, Faculty of Medicine Tottori University Yonago Japan; ^3^ Department of Family Medicine, General Practice and Community Health, Institute of Medicine University of Tsukuba Tsukuba Japan; ^4^ Department of Food and Nutrition Koriyama Women's University Koriyama Japan; ^5^ Department of Hygiene and Preventive Medicine Fukushima Medical University Fukushima Japan; ^6^ National Institute of Alcoholism Kurihama National Hospital Yokosuka Japan; ^7^ Department of Food Science and Nutrition, Faculty of Life and Environmental Science Showa Women's University Tokyo Japan; ^8^ Division of Public Health, Department of Social Medicine Nihon University School of Medicine Tokyo Japan; ^9^ Department of Public Health, School of Medicine International University of Health and Welfare Narita Japan

**Keywords:** adolescent, alcohol drinking, COVID‐19, Japan, lifestyle

## Abstract

**Background:**

The COVID‐19 pandemic may have influenced drinking behaviors in minors by disrupting daily routines and increasing psychosocial stress, although alcohol use among Japanese adolescents has declined in recent years. We aimed to clarify the relationships between current alcohol consumption and lifestyle factors during the COVID‐19 pandemic based on a nationwide cross‐sectional survey.

**Methods:**

This cross‐sectional study analyzed data from the 2021 Lifestyle Survey of Adolescents, a nationwide survey conducted in Japan during the COVID‐19 pandemic. A total of 15 549 junior and senior high school students (7645 boys and 7904 girls) were included. Current alcohol consumption was defined as drinking on at least 1 day in the past 30 days. Multivariable logistic regression analyses were used to examine associations between current alcohol consumption and lifestyle factors, including irregular sleep patterns, irregular dietary habits, and increased screen time. Sex‐stratified analyses and interaction tests were also performed.

**Results:**

The overall prevalence of current alcohol consumption was 2.1%, with slightly higher rates among boys (2.2%) than girls (2.0%). Current alcohol consumption was significantly associated with irregular sleep patterns (odds ratio [OR] = 1.51; 95% confidence interval [CI], 1.17–1.95) and irregular dietary habits (OR = 1.68; 95% CI, 1.18–2.40). An association with increased screen time was also observed (OR = 1.29; 95% CI, 1.00–1.69), particularly among boys. A significant interaction by sex was detected for irregular sleep patterns (*p* for interaction = 0.013).

**Conclusions:**

Alcohol consumption among Japanese adolescents was associated with irregular sleep and dietary habits and, among boys, with increased screen time. These findings highlight the importance of promoting regular routines and addressing lifestyle‐related risks to prevent current alcohol consumption among adolescents during public health crises.

## Abstract 2

Nationwide survey of Japanese adolescents during the COVID‐19 pandemic (*n* = 15 549) showed associations of current alcohol consumption with irregular sleep patterns (OR 1.51), irregular dietary habits (OR 1.68), and increased screen time (OR 1.29). The association with irregular sleep patterns was stronger in girls (OR 1.84; *p* for interaction = 0.013).

## Graphical abstract

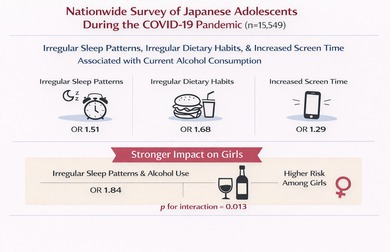

## 1 Introduction

Underage drinking remains a significant public health concern because of its association with a wide range of physical, psychological, and social consequences. Early initiation of alcohol consumption increases the likelihood of developing problematic drinking patterns or dependence in young adulthood [1, 2, 3]. Although adolescent drinking rates vary internationally, many countries, including Japan, have reported a recent decline [4, 5, 6]. In Japan, the national health policy Health Japan 21 (the Third Term) maintains a target of eliminating alcohol consumption among those aged < 20 years and reports a prevalence of 2.2% [7].

Despite these improvements, underage drinking persists among some adolescents and is influenced by multiple behavioral and environmental factors. Previous studies have identified associations with irregular dietary habits [8, 9, 10], irregular sleep patterns [11, 12], and psychosocial influences such as parental alcohol use and peer relationships [13]. Evidence suggests that sleep disturbances and irregular routines are associated with adolescent alcohol consumption [14, 15], potentially through mechanisms such as emotional dysregulation and stress. The relationship may also be bidirectional, as alcohol consumption itself can impair sleep quality and patterns.

The COVID‐19 pandemic has caused widespread disruptions in adolescents' daily lives. In Japan, the study period (May–August 2021) coincided with the spread of the Delta variant and ongoing social restrictions, including school closures and the suspension of extracurricular activities. Various studies have reported pandemic‐related changes in adolescents' lifestyles, including alterations in sleep, diet, physical activity, screen time, and mental health [16, 17]. These disruptions may also have influenced drinking behaviors by destabilizing daily routines and increasing psychosocial stress.

While several Japanese studies—including those conducted by our group—have examined individual lifestyle factors associated with adolescent alcohol consumption [18, 19, 20, 21], few have comprehensively assessed these factors within a single analytical framework. To the best of our knowledge, no previous nationwide study has investigated the associations between current alcohol consumption among adolescents and lifestyle factors during the COVID‐19 pandemic.

To address this gap, we analyzed data from a 2021 nationwide survey of Japanese adolescents to examine the prevalence of current alcohol consumption and its associations with pandemic‐related lifestyle factors. Our primary hypothesis was that irregular sleep patterns would be associated with higher odds of current alcohol consumption, with a stronger association anticipated among girls. As secondary hypotheses, we examined whether irregular dietary habits and increased screen time were also associated with current alcohol consumption.

## 2 Methods

### 2.1 Study Design and Participants

Adolescent drinking behaviors in Japan have previously been reported by our group using nationwide surveys [18, 19, 20, 21]. This study analyzed data from the 2021 Lifestyle Survey of Adolescents, which has been conducted periodically since 1996 with support from the Ministry of Health, Labour and Welfare and is overseen by our working group. A single‐stage stratified cluster sampling method was employed to obtain a nationally representative sample of Japanese adolescents, consistent with previous surveys [15, 18, 19, 20]. The sampling unit was the school, and schools were randomly selected from regional blocks across the country [15].

The cross‐sectional nationwide survey was conducted from May to August 2021, i.e., during the COVID‐19 pandemic, and targeted students enrolled in full‐time junior and senior high schools in Japan. Based on the 2020 national school registry, 91 junior and 62 senior high schools were selected using a regionally stratified cluster random sampling method. Of these, 64 junior and 42 senior high schools were randomly assigned to the web‐based survey, and 27 junior and 20 senior high schools were assigned to the paper‐based survey. The target population included students in grades 7 through 12.

**FIGURE 1:**
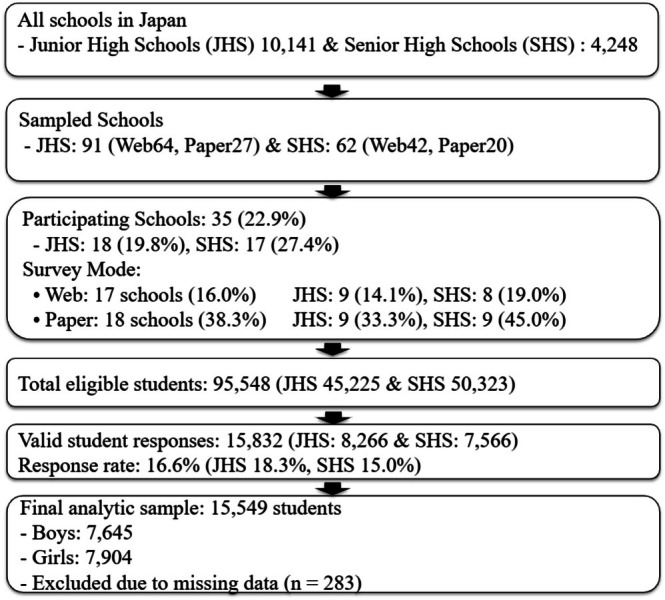
Flowchart of the procedure for selecting eligible respondents in this study.

Ultimately, 18 junior high schools (response rate: 19.8%) and 17 senior high schools (27.4%) participated, resulting in a total of 35 schools and an overall response rate of 22.9%. Of these, 17 schools (16.0%) participated in the web‐based survey, and 18 schools (38.3%) participated in the paper‐based survey (see Figure 1 for details).

The survey was distributed to 95 548 students, of whom 15 832 (8266 from junior and 7566 from senior high schools) provided valid responses. The overall valid response rate was 16.6% (18.3% for junior and 15.0% for senior high school students). After excluding 283 students with missing data on key variables such as drinking status, sex, grade, and COVID‐19‐related items, the final analytic sample included 15 549 students (7645 boys and 7904 girls), which was used for the descriptive analyses.

### 2.2 Survey Procedure and Ethical Considerations

For the paper‐based survey, request letters were sent to school principals, and sealed questionnaires were distributed to students. Teachers explained the study's purpose, the voluntary nature of participation, and the assurance of anonymity, emphasizing that responses would not be disclosed to school staff or parents. Prior to the survey, explanatory documents were distributed to parents or guardians through the schools. Because our survey included both web‐based and paper‐based formats, potential differences in response patterns were considered. A previous study using the same nationwide Lifestyle Survey of Adolescents dataset reported differences in participant characteristics between survey modes, although the main associations were broadly consistent across modes [21]. Therefore, additional analyses stratified by survey mode were conducted, and the results are presented in the Supporting Information.

In accordance with the Ethical Guidelines for Medical and Health Research Involving Human Subjects in Japan, informed consent was obtained from each high school student and from the parent or guardian of each junior high school student. For the paper‐based survey, completion and return of the questionnaire were regarded as implied consent. For the web‐based survey, students were required to check a consent box before proceeding to the questionnaire. Explanatory documents were also distributed to parents or guardians through the schools. Participation was voluntary, and students or their guardians could opt out by not completing the survey.

This study was part of the ongoing Lifestyle Survey of Adolescents, supported by the Ministry of Health, Labour and Welfare, and utilized the same dataset reported by Yoshida et al. [21] The survey protocol was approved by the Ethics Committee of the Faculty of Medicine, Tottori University (Approval No. 20A099, approved April 30, 2021) and the Ethics Committee of Okayama University (Approval No. K2108‐042, approved August 13, 2021).

### 2.3 Measures

The selection and definitions of measures, including current alcohol consumption (defined as drinking on at least 1 day in the past 30 days), were informed by our group's previous studies on adolescent health‐related behaviors in Japan [6, 15, 18, 19, 20, 21]. Demographic variables included sex, grade, school type, and school location. School location was categorized into three geographic regions: Eastern Japan (Hokkaido, Tohoku, and Kanto), Central Japan (Hokuriku, Koshinetsu, and Tokai), and Western Japan (Kansai, Chugoku, Shikoku, Kyushu, and Okinawa). Lifestyle‐related variables included current smoking, bedtime, enjoyment of school life, parental alcohol use, and depressed mood.

Current smoking was defined as tobacco use on at least 1 day in the past 30 days. Bedtime was categorized as either “before midnight” or “after midnight.” Enjoyment of school life was assessed with the response options “yes,” “neutral,” or “no,” and treated as a three‐category variable.

Parental alcohol use was assessed with the question: “Have you ever felt uncomfortable when your parent(s) drank alcohol?” Response options were “yes,” “no,” and “my parent(s) do not drink.” Respondents who answered “yes” or “no” were classified as having drinking parents, while those who answered “my parent(s) do not drink” were classified as having nondrinking parents.

Depressed mood was assessed with the question: “In the past 30 days, have you felt more down or depressed than usual?” Responses were categorized into four levels: (1) never, (2) rarely, (3) sometimes, and (4) often. For analysis, responses (1) and (2) were recoded as “No” and (3) and (4) as “Yes.”

Perceived negative impacts of the COVID‐19 pandemic were assessed using a multiple‐response question: “Due to the COVID‐19 pandemic in 2020, including school closures and other unusual circumstances, did you experience any negative effects? Please check all that apply.” For the present analysis, seven relevant items were chosen based on conceptual importance and prevalence: irregular sleep patterns, irregular dietary habits, difficulty studying, increased screen time, accumulated stress, physical inactivity, and none of the above. Each item was treated as a separate binary variable (checked = Yes, unchecked = No) in the statistical analysis. In the questionnaire, “irregular dietary habits” referred to disruptions in eating patterns, including changes in meal frequency, timing, or nutritional balance. In this study, “increased screen time” referred to spending more time on gaming or internet use and did not include educational screen use such as online classes.

### 2.4 Statistical Analysis

Analyses began with the 15 549 students after applying the exclusion criteria described above. Participants with missing data on key variables (current alcohol consumption, sex, grade, and COVID‐19‐related items) were excluded. Ultimately, 15 549 students were included in the descriptive analyses. For the multivariable logistic regression analyses, a complete‐case approach was applied, excluding participants with missing values on any covariates in the model. The final analytic sample comprised 15 351 students. The numbers of missing values for each covariate were as follows: bedtime (*n* = 9), enjoyment of school life (*n* = 61), current smoking (*n* = 127), parental alcohol use (*n* = 59), and depressed mood (*n* = 24). Because some participants had more than one missing value, a total of 198 participants were excluded. These exclusions accounted for 1.3% of the total sample, a proportion too small to materially influence the findings.

Differences in distributions were evaluated using chi‐square tests. To examine the associations between lifestyle changes during the COVID‐19 pandemic and current alcohol consumption, chi‐square tests were first conducted to compare the prevalence of each lifestyle factor by current alcohol consumption status. To account for the complex survey design, including school‐level clustering and sampling weights, all analyses were survey‐weighted. Multivariable logistic regression analyses were then performed to estimate odds ratios (ORs) and 95% confidence intervals (CIs) for the associations between lifestyle factors and current alcohol consumption. The models were adjusted for sex, grade level, and school location, which were selected based on prior research identifying these as important demographic factors associated with adolescent drinking behaviors. Additional covariates—enjoyment of school life, current smoking, and parental alcohol use—were included based on their significant associations with current alcohol consumption in the univariate analyses of the present study (Table 1). Bedtime and depressed mood also showed significant univariate associations but were excluded from the final multivariable models to avoid multicollinearity and overadjustment due to conceptual overlap with the main explanatory variables (irregular sleep patterns and accumulated stress, respectively). To examine sex differences, analyses were stratified by sex, and interaction tests using multiplicative terms were conducted. In addition, exploratory analyses stratified by school level (junior vs. senior high school) were conducted.

**TABLE 1:** Demographic and behavioral characteristics of participants by sex and current alcohol consumption status during the COVID‐19 pandemic.

	Current alcohol consumption (All, *n* = 15 549)				Current alcohol consumption (Boys, *n* = 7645)				Current alcohol consumption (Girls, *n* = 7904)			
	(−), *n* = 15 219		(+), *n* = 330		(−), *n* = 7474		(+), *n* = 171		(−), *n* = 7745		(+), *n* = 159	
	*n*	% (95% CI)	*n*	% (95% CI)	*n*	% (95% CI)	*n*	% (95% CI)	*n*	% (95% CI)	*n*	% (95% CI)
Type of school												
Junior high school	7959	44.4 (25.0–65.8)	128	34.7 (17.8–56.7)	4260	49.1 (27.1–71.6)	79	40.8 (21.6–63.1)	3699	39.7 (21.4–61.4)	49	28.0 (12.2–51.9)
Senior high school	7260	55.6 (34.2–75.0)	202	65.3 (43.3–82.2)	3214	50.9 (28.4–72.9)	92	59.2 (36.9–78.4)	4046	60.3 (38.6–78.6)	110	72.0 (48.1–87.8)
Grades												
7th	2702	14.9 (9.0–23.8)	37	9.8 (5.3–17.3)	1438	16.4 (9.9–25.9)	28	14.3 (7.7–24.9)	1264	13.4 (7.8–22.1)	9	4.6 (2.2–9.4)
8th	2717	15.1 (9.1–24.0)	37	9.9 (5.3–17.7)	1412	16.1 (9.6–25.6)	28	14.3 (7.7–24.8)	1305	14.1 (8.0–23.7)	9	5.0 (2.3–10.5)
9th	2540	14.4 (8.5–23.1)	54	15.1 (7.7–27.3)	1410	16.6 (9.6–27.2)	23	12.2 (6.2–22.5)	1130	12.1 (7.0–20.2)	31	18.4 (7.8–37.4)
10th	2891	22.0 (14.7–31.6)	43	14.4 (7.9–24.7)	1272	19.8 (12.3–30.2)	14	9.0 (4.5–17.1)	1619	24.2 (16.7–33.8)	29	20.4 (11.4–33.9)
11th	2186	16.7 (10.6–25.4)	83	25.8 (16.1–38.6)	969	15.4 (8.9–25.5)	37	23.4 (14.3–35.8)	1217	18.0 (11.8–26.4)	46	28.5 (17.1–43.6)
12th	2183	16.9 (10.7–25.6)	76	25.1 (16.8–35.7)	973	15.6 (9.1–25.5)	41	26.8 (17.0–39.8)	1210	18.1 (11.9–26.5)	35	23.1 (14.2–35.2)
School location												
Eastern Japan	6220	45.9 (25.4–67.8)	119	39.6 (20.2–62.9)	2945	45.0 (23.2–68.9)	55	35.7 (17.0–60.1)	3275	46.7 (26.0–68.7)	64	44.0 (21.1–69.8)
Central Japan	4973	30.1 (15.1–52.9)	90	28.7 (13.1–51.8)	2551	31.0 (15.3–54.9)	57	34.5 (16.5–58.5)	2422	29.8 (14.1–52.4)	33	22.1 (8.6–46.0)
Western Japan	4026	23.2 (10.5–43.7)	121	31.7 (14.5–55.9)	1978	23.0 (10.0–44.6)	59	29.7 (13.8–52.8)	2048	23.4 (10.7–43.7)	62	34.0 (13.8–62.2)
Bedtimes												
Before midnight	9879	61.8 (54.6–68.5)	167	48.0 (39.6–56.6)	5073	64.9 (56.9–72.1)	92	50.3 (38.6–62.0)	4806	58.7 (51.9–65.3)	75	45.5 (36.3–55.0)
After midnight	5331	38.2 (31.5–45.4)	163	52.0 (43.4–60.4)	2398	35.1 (27.9–43.1)	79	49.7 (38.0–61.4)	2933	41.3 (34.7–48.1)	84	54.5 (45.0–63.7)
Enjoyment of school life												
Yes	10 276	68.2 (64.4–71.8)	176	54.8 (44.8–64.4)	5284	71.1 (67.3–74.6)	96	59.4 (47.9–70.0)	4992	65.3 (61.2–69.2)	80	49.6 (38.2–61.0)
Neither	3967	25.9 (23.2–28.7)	98	29.4 (23.7–35.9)	1735	23.2 (20.7–26.0)	46	25.7 (18.6–34.2)	2232	28.5 (25.5–31.7)	52	33.6 (26.3–41.8)
No	918	6.0 (5.1–7.0)	53	15.8 (10.7–22.7)	424	5.7 (4.7–6.9)	26	14.9 (8.1–26.0)	494	6.2 (5.2–7.4)	27	16.8 (11.1–24.6)
Current smoking												
No	15 033	99.6 (99.4–99.7)	279	86.1 (79.7–90.7)	7361	99.5 (99.2–99.7)	148	88.3 (81.4–92.8)	7672	99.7 (99.5–99.8)	131	83.6 (75.6–89.4)
Yes	64	0.4 (0.31–0.57)	46	13.9 (9.3–20.3)	40	0.5 (0.3–0.7)	21	11.7 (7.2–18.6)	24	0.3 (0.2–0.5)	25	16.4 (10.6–24.4)
Parental alcohol use												
No	2056	13.3 (12.1–14.6)	15	4.4 (2.4–7.8)	989	13.0 (11.6–14.5)	8	4.6 (1.8–10.9)	1067	13.7 (12.4–15.1)	7	4.1 (2.0–8.4)
Yes	13 105	86.7 (85.4–87.9)	314	95.6 (92.2–97.6)	6446	87.0 (85.5–88.4)	163	95.4 (89.1–98.2)	6659	86.3 (84.9–87.6)	151	95.9 (91.6–98.0)
Depressed mood (past 30 days)												
No	8177	52.7 (49.9–55.4)	142	43.0 (34.6–51.9)	4616	60.2 (56.6–63.7)	95	55.4 (45.6–64.7)	3561	45.1 (42.3–48.0)	47	29.0 (21.4–38.0)
Yes	7019	47.3 (44.6–50.1)	187	57.0 (48.1–65.4)	2851	39.8 (36.3–43.4)	76	44.6 (35.3–54.4)	4168	54.9 (52.0–57.7)	111	71.0 (62.0–78.6)

*Note:* Current alcohol consumption was defined as drinking alcohol on at least 1 day in the past 30 days. Current smoking was defined as tobacco use on at least 1 day in the past 30 days. Values are presented as observed frequencies (*n*) and weighted percentages with 95% confidence intervals (95% CIs). Percentages were estimated using survey sampling weights accounting for the one‐stage cluster sampling design with unequal cluster sizes.

Model fit was assessed using the Hosmer–Lemeshow goodness‐of‐fit test. Multicollinearity was evaluated using variance inflation factors (VIFs), and no serious multicollinearity was detected in the final models. Additionally, conceptual relevance and potential overlap among variables were also carefully considered during the construction of the models. All analyses were performed using Stata/SE version 18.5 (StataCorp LLC, College Station, TX, USA). A two‐tailed significance level of *p* < 0.05 was considered statistically significant. ORs and 95% CIs were calculated and rounded to two decimal places. Because the lifestyle variables were selected based on a priori hypotheses and conceptual relevance, we did not apply formal corrections for multiple comparisons. This approach is commonly used in epidemiological studies examining interrelated lifestyle factors. Detailed variable definitions, recoding procedures, and analytic workflows are provided in the Supporting Information to facilitate reproducibility.

## 3 Results

Data from 15 549 students (7645 boys and 7904 girls) were included in the final analysis after excluding 283 respondents with missing data on key variables, such as current alcohol consumption status and sex. The analytic sample comprised students from 18 junior high schools and 17 senior high schools that participated in the nationwide survey conducted between May and August 2021. The overall valid response rate was 16.6%, with rates of 18.3% for junior high school students and 15.0% for senior high school students.

Table 1 presents the demographic characteristics of participants, stratified by sex and current alcohol consumption status. Current alcohol consumption was more prevalent among students in higher grade levels, those attending senior high school, those residing in Western Japan, those with later bedtimes, those reporting lower enjoyment of school, current smokers, those with parental alcohol use, and those reporting depressed mood (“Yes”) within the past 30 days.

The overall prevalence of current alcohol consumption was 2.1%. By sex, 2.2% (*n* = 171) of boys and 2.0% (*n* = 159) of girls were identified as current drinkers. By school level, the prevalence was 1.6% among junior high school students (1.9% in boys and 1.3% in girls) and 2.7% among senior high school students (2.8% in boys and 2.7% in girls).

Table 2 summarizes the distribution of self‐reported lifestyle changes during the COVID‐19 pandemic and their associations with current alcohol consumption status. Across most categories, current drinkers reported a higher frequency of negative behavioral changes than non‐drinkers. For example, irregular sleep patterns were more frequently reported by current drinkers than by non‐drinkers (boys: 28.9% [95% CI, 20.7–38.7] vs. 22.7% [95% CI, 19.7–25.9]; girls: 49.4% [95% CI, 42.3–56.4] vs. 31.6% [95% CI, 28.8–34.6]). Similarly, irregular dietary habits were more common among drinkers (boys: 11.1% [95% CI, 6.4–18.5] vs. 7.3% [95% CI, 6.2–8.6]; girls: 28.1% [95% CI, 20.2–37.6] vs. 15.4% [95% CI, 14.0–17.0]).

**TABLE 2:** Associations between lifestyle factors and current alcohol consumption among Japanese adolescents during the COVID‐19 pandemic.

	Current alcohol consumption (All, *n* = 15 549)				Current alcohol consumption (Boys, *n* = 7645)				Current alcohol consumption (Girls, *n* = 7904)			
	(−), *n* = 15 219		(+), *n* = 330		(−), *n* = 7474		(+), *n* = 171		(−), *n* = 7745		(+), *n* = 159	
	*n*	% (95% CI)	*n*	% (95% CI)	*n*	% (95% CI)	*n*	% (95% CI)	*n*	% (95% CI)	*n*	% (95% CI)
None	4279	27.3 (24.8–30.0)	89	26.5 (20.3–33.7)	2570	33.4 (30.5–36.7)	56	32.4 (24.7–41.1)	1709	21.3 (18.0–25.0)	33	19.8 (13.3–28.5)
Irregular sleep patterns	4014	27.2 (24.6–29.9)	123	38.5 (31.9–45.5)	1632	22.7 (19.7–25.9)	46	28.9 (20.7–38.7)	2382	31.6 (28.8–34.6)	77	49.4 (42.3–56.4)
Irregular dietary habits	1721	11.4 (10.1–12.8)	63	19.1 (13.9–25.7)	553	7.3 (6.2–8.6)	21	11.1 (6.4–18.5)	1168	15.4 (14.0–17.0)	42	28.1 (20.2–37.6)
Difficulty studying	3471	23.6 (20.6–26.8)	82	25.0 (18.6–32.7)	1514	21.0 (18.0–24.4)	36	20.2 (12.2–31.7)	1957	26.1 (22.7–29.8)	46	30.3 (22.6–39.4)
Increased screen time	7040	46.7 (44.6–48.8)	161	49.3 (43.4–55.2)	3217	43.6 (41.5–45.7)	83	49.1 (40.1–58.2)	3823	49.9 (46.8–52.9)	78	49.5 (43.0–56.0)
Accumulated stress	2452	16.1 (14.6–17.9)	56	15.8 (12.4–19.9)	1095	14.6 (12.9–16.6)	23	12.3 (8.0–18.4)	1357	17.6 (15.8–19.6)	33	19.7 (14.4–26.4)
Physical inactivity	6669	43.9 (41.9–45.9)	140	43.8 (36.8–51.1)	2645	35.6 (33.8–37.4)	55	33.6 (27.3–40.7)	4024	52.1 (49.0–55.1)	85	55.3 (44.6–65.6)

*Note:* Current alcohol consumption was defined as drinking alcohol on at least 1 day in the past 30 days. Values are presented as observed frequencies (*n*) and weighted percentages with 95% confidence intervals (95% CIs). Percentages were estimated using survey sampling weights accounting for the one‐stage cluster sampling design with unequal cluster sizes.

Based on complete data from 15 351 students, multivariable logistic regression analyses were conducted to examine the associations between lifestyle changes and current alcohol consumption (Table 3). Irregular sleep patterns were associated with current alcohol consumption in the overall sample (OR = 1.51; 95% CI, 1.17–1.95). In sex‐stratified analyses, the association was stronger in girls (OR = 1.84; 95% CI, 1.34–2.51) than in boys (OR = 1.24; 95% CI, 0.87–1.78), with a significant interaction (*p* for interaction = 0.013).

**TABLE 3:** Sex‐stratified analyses of associations between lifestyle factors and current alcohol consumption during the COVID‐19 pandemic.

	All (*n* = 15 351)	Boys (*n* = 7533)	Girls (*n* = 7818)	*p* for interaction
	OR (95% CI)	OR (95% CI)	OR (95% CI)	
Irregular sleep patterns	1.51 (1.17–1.95)**	1.24 (0.87–1.78)	1.84 (1.34–2.51)***	0.013
Irregular dietary habits	1.68 (1.18–2.40)**	1.49 (0.87–2.54)	1.77 (1.11–2.82)*	0.408
Difficulty studying	1.04 (0.72–1.52)	0.93 (0.51–1.71)	1.16 (0.75–1.82)	0.390
Increased screen time	1.29 (1.00–1.69)	1.47 (1.01–2.13)*	1.13 (0.82–1.54)	0.289
Accumulated stress	0.92 (0.68–1.24)	0.72 (0.42–1.23)	1.18 (0.79–1.75)	0.165
Physical inactivity	1.14 (0.87–1.49)	1.11 (0.78–1.56)	1.17 (0.79–1.73)	0.691

*Note:* Models were adjusted for sex, grade level, school location, enjoyment of school life, current smoking, and parental alcohol use. Current alcohol consumption was defined as drinking alcohol on at least 1 day in the past 30 days. Odds ratios (ORs) and 95% confidence intervals (CIs) were estimated using survey‐weighted logistic regression models and rounded to two decimal places.

* *p* < 0.05.

** *p* < 0.01.

*** *p* < 0.001.

Irregular dietary habits were also associated with current alcohol consumption in the overall sample (OR = 1.68; 95% CI, 1.18–2.40). This association was present in both sexes: among boys (OR = 1.49; 95% CI, 0.87–2.54) and among girls (OR = 1.77; 95% CI, 1.11–2.82). Additionally, increased screen time was associated with current alcohol consumption in the overall sample (OR = 1.29; 95% CI, 1.00–1.69) and among boys (OR = 1.47; 95% CI, 1.01–2.13); however, no significant interaction by sex was observed for this factor.

The Hosmer–Lemeshow tests indicated acceptable model fit for the sex‐stratified models and no substantial misfit in the overall model. Sensitivity analyses additionally adjusting for bedtime and depressed mood yielded results consistent with the primary analyses (Table S1). The results of exploratory stratified analyses by school level are presented in Table S2, and the results of analyses stratified by survey mode (web‐based vs. paper‐based) are presented in Table S3.

## 4 Discussion

Our findings indicate that current alcohol consumption among adolescents is associated with several lifestyle‐related factors, particularly irregular sleep patterns and dietary habits. The association between sleep patterns and current alcohol consumption was notably stronger among girls, as evidenced by a significant interaction. Associations with irregular dietary habits and increased screen time were also observed, with some sex‐specific differences. Derived from a nationwide study of Japanese junior and senior high school students during the COVID‐19 pandemic, these findings highlight the importance of lifestyle behaviors in understanding adolescent alcohol consumption. The overall prevalence (2.1%) was essentially consistent with the 2.2% reported in Health Japan 21 (the Third Term) [7], with minor differences attributable to analytic criteria. Although the overall prevalence of current alcohol consumption was relatively low, the presence of multiple associated factors underscores the need for targeted prevention strategies that take sex differences into account.

Irregular sleep patterns were associated with current alcohol consumption, with a particularly strong association among girls (*p* for interaction = 0.013). This finding suggests that adolescent girls may be more vulnerable to the adverse effects of sleep disruption. Erol and Karpyak have noted that sex and gender‐related factors—such as differences in alcohol metabolism, hormonal responses, and psychosocial stress—can heighten females' sensitivity to alcohol‐related harms [22]. These biological and psychosocial mechanisms may partly explain the stronger association observed among girls in our study. Previous studies have also shown that sleep disturbances during adolescence increase psychological stress and impair self‐regulation, thereby facilitating risk behaviors [14, 15, 18]. Furthermore, girls are more likely than boys to experience depressive and anxiety symptoms during adolescence, particularly under stressful circumstances such as the COVID‐19 pandemic [16], both of which are linked to alcohol‐related issues. This aligns with earlier longitudinal findings demonstrating that depressive symptoms in adolescence predict later alcohol‐related problems [1]. A recent meta‐analysis also supports the link between adolescent sleep deprivation and increased alcohol‐related risk behaviors [23]. Although “accumulated stress” was not associated with current alcohol consumption in our analysis, this single self‐reported item may not have fully captured the broader spectrum of psychological distress, such as anxiety, depressive symptoms, or chronic emotional burden. It is also possible that stress influences alcohol use indirectly through disrupted sleep or impaired self‐regulation rather than through a direct pathway. These factors may partly explain why the association between stress and alcohol use was not evident in our study despite the stronger sleep–alcohol association observed among girls. Although bedtime (before/after midnight) was excluded from the final multivariable model due to multicollinearity with irregular sleep patterns, we prioritized the subjective measure because it more comprehensively captured disruptions in sleep regularity—including variability in timing, irregular sleep–wake routines, and day–night reversal—which were conceptually relevant to pandemic‐related lifestyle changes.

These findings support a sex‐specific interpretation of the association between irregular sleep patterns and current alcohol consumption. Additionally, regional surveys conducted in Japan during the COVID‐19 pandemic reported shorter sleep duration, reduced physical activity, and increased depressive symptoms among girls [24, 25], consistent with our findings. Our findings are broadly consistent with prior evidence linking disrupted sleep with adolescent risk‐related behaviors. A recent meta‐analysis by Short et al. also reported an association between insufficient sleep and risk‐taking behaviors in adolescents [23]. However, direct quantitative comparisons across studies should be interpreted cautiously because the outcomes examined, definitions, and measurement methods differ and the meta‐analysis did not focus specifically on alcohol use.

Irregular dietary habits were also associated with current alcohol consumption. Specifically, breakfast skipping and other irregular eating patterns have been linked to a higher likelihood of engaging in risky behaviors, including alcohol consumption [8, 9, 10], and our findings support this association, underscoring the potential importance of dietary habits in prevention efforts. Breakfast skipping not only reflects poor nutrition but may also indicate disrupted routines, family‐related stress, or socioeconomic adversity. Previous research has reported that adolescents facing food insecurity, limited parental supervision, or emotional distress are more likely to skip meals, all of which are associated with an increased risk of engaging in health‐risk behaviors, including alcohol use [10]. Furthermore, pandemic‐related restrictions such as school closures and reduced opportunities for social interaction may have exacerbated adolescents' eating patterns, potentially amplifying these risk behaviors [17].

Increased screen time was also linked to current alcohol consumption, particularly among boys. This association may reflect multiple factors, including reduced parental supervision and greater engagement with online peer communities. Although these aspects were not directly assessed in our study, previous nationwide research has shown that problematic internet use is associated with adolescent drinking behavior [20]. These findings suggest that changes in family dynamics and social environments during the pandemic may have indirectly contributed to underage drinking. A study of Japanese high school boys reported increased screen time and decreased social satisfaction during the pandemic [25], supporting our findings. Although the association was more pronounced among boys, there was no significant interaction by sex, suggesting that increased screen time may also be relevant to girls. Nationwide studies in adults have similarly reported that pandemic‐related psychological distress was associated with increased alcohol use and reduced physical activity [26], implying that comparable mechanisms may operate among adolescents.

To the best of our knowledge, this is one of the few nationwide investigations to assess associations between lifestyle behaviors and underage drinking under the unique societal conditions of the COVID‐19 pandemic in Japan. Although the pandemic was a global phenomenon, the societal and policy responses in Japan—such as relatively short school closures and limited legal enforcement of lockdowns—may have shaped adolescents' lifestyles differently than in other countries.

Notably, a nationwide mobility analysis during the COVID‐19 pandemic in Japan demonstrated that even non‐compulsory public health measures, such as state‐of‐emergency declarations and voluntary restrictions, led to significant reductions in population mobility, including decreased visits to recreational areas and increased time spent at home [27]. This suggests that adolescents' lifestyle changes may have been substantially influenced by these broad, population‐level behavioral shifts, despite the absence of legally enforced lockdowns.

Unlike many Western countries that adopted strict lockdowns with legal penalties, Japan relied primarily on voluntary behavioral restrictions, including recommendations to avoid the “Three Cs” (closed spaces, crowded places, and close‐contact settings) without mandatory stay‐at‐home orders [28].

Given that such lifestyle disruptions may recur during future public health crises or environmental changes, our findings may help inform the development of robust and targeted prevention strategies.

Several limitations should be acknowledged. First, the cross‐sectional design precludes any conclusions about causality. Additionally, the observed relationships may be bidirectional; for example, irregular sleep may increase vulnerability to drinking, while alcohol use may also disrupt sleep–wake rhythms. Second, the use of self‐reported data may introduce recall bias or social desirability bias, particularly underreporting of alcohol use given the legal age limit and the anonymous survey format. Because underage drinking is illegal in Japan, underreporting is likely; such nondifferential misclassification may have biased the associations toward the null. Moreover, all lifestyle‐related variables—including sleep patterns, dietary habits, and screen time—were assessed using subjective self‐reports without objective or quantitative measures, which may have introduced measurement error due to individual differences in interpretation. Third, the sample may not be fully representative, limiting the generalizability of the findings. In particular, the overall school participation rate (22.9%) and student response rate (16.6%) were relatively low, raising the possibility of nonresponse bias. The participation rate was especially low in the web‐based survey (16.0%), suggesting that students or schools with different health behaviors or administrative priorities may have opted out. Although regionally stratified sampling was employed, participating schools may have differed systematically—for example, in their emphasis on health education or willingness to engage in school‐based surveys—which could have influenced the observed prevalence and associations. Fourth, unmeasured confounding variables—such as household income, academic performance, access to alcohol, or other social determinants of health—may have influenced the observed associations. Fifth, because this study relied on a single cross‐sectional survey conducted during the COVID‐19 pandemic, we were unable to examine temporal trends or compare adolescent drinking behaviors across different pandemic phases (e.g., pre‐, during‐, and post‐COVID‐19 periods). Longitudinal or repeated cross‐sectional surveys would be necessary to evaluate such changes over time.

The findings of this study suggest that preventive strategies incorporating school‐based education, behavioral interventions, and family engagement may be effective in promoting regular sleep patterns and healthy dietary habits among adolescents. In particular, programs designed to foster consistent daily routines, support balanced eating habits, and manage excessive screen time could help reduce the risk of underage drinking. Tailoring these interventions to reflect sex and school‐level differences identified in this study may further enhance their effectiveness. To better understand the causal pathways linking lifestyle behaviors and adolescent alcohol use, future research should adopt longitudinal and intervention‐based designs. Examining how pandemic‐related disruptions interact with the developmental vulnerabilities of adolescence may also help guide the creation of more flexible and responsive prevention strategies for future societal or environmental challenges.

## 5 Conclusions

Building on these findings, this study highlights how adolescent drinking behavior may be associated with key lifestyle factors during the COVID‐19 pandemic, offering valuable insights to inform prevention efforts during future societal crises. Promoting regular sleep patterns, encouraging healthy dietary habits, and monitoring changes in screen time could play an important role in reducing underage drinking. Although causal relationships cannot be inferred due to the cross‐sectional design, the results suggest that certain subgroups of adolescents—particularly girls—may be more vulnerable, with multiple lifestyle‐related risks potentially clustering under pandemic‐related disruptions. Future educational and public health initiatives should incorporate integrated, sex‐sensitive strategies that address these vulnerabilities in adolescent populations. Given that infectious disease outbreaks can substantially disrupt adolescents' daily routines, greater attention to lifestyle changes during such periods may help promote healthier behaviors and prevent the onset of harmful ones.

## Author Contributions

Conceptualization: M.N., K.Y., H. Kanda, and T.H. Methodology: M.N., K.Y., H. Kanda, and T.H. Formal analysis: M.N., K.Y., H. Kanda, and T.H. Data curation: Y. Kuwabara, A.K., H.Y., R.M., H.M., H. Kim, A.I. Writing – original draft preparation: M.N., H. Kanda, and T.H. Writing – review and editing: O.I., Y. Otsuka, M.J., H. Kasuga, T. Ito, and Y. Osaki. Supervision: H. Kanda, Y. Kaneita, S.H., and Y. Osaki. Project administration: Y. Osaki. All authors approved the final manuscript.

## Funding

This research was supported by a grant for Comprehensive Research on Lifestyle‐Related Diseases, including Cardiovascular Diseases and Diabetes Mellitus, from the Ministry of Health, Labour and Welfare, Health Science Research Fund in Japan (grant no. 20FA1003). The funder had no role in the design of the study; in the collection, analyses, or interpretation of data; in the writing of the manuscript; or in the decision to publish the results.

## Ethics Statement

Approved by the Ethics Committee of the Faculty of Medicine, Tottori University (Approval No. 20A099, April 30, 2021) and the Ethics Committee of Okayama University (Approval No. K2108‐042, August 13, 2021).

## Consent

Informed consent was obtained from all participants and their guardians, as appropriate, in accordance with the Ethical Guidelines for Medical and Health Research Involving Human Subjects in Japan.

## Conflicts of Interest

H.Y. received research funding from Asahi Breweries and Sanwa Shurui, which was unrelated to the conduct of this study. H.M. received speaking fees from Otsuka Pharmaceutical and Nippon Shinyaku, also unrelated to the current study. H. Kanda received research funding from the Osake‐no‐Kagaku Foundation, unrelated to this research. The remaining authors declare no conflicts of interest.

## Supporting information


**Data S1:** npr270089‐sup‐0001‐Supinfo.docx.

## Data Availability

Due to restrictions imposed by the ethics committees, individual‐level data are not publicly available. Secondary use requires approval from the ethics committee of the corresponding author's institution, and requests should be addressed to the corresponding author.

## References

[1] N. R. Marmorstein , “Longitudinal Associations Between Alcohol Problems and Depressive Symptoms: Early Adolescence Through Early Adulthood,” Alcoholism, Clinical and Experimental Research 33, no. 1 (2009): 49–59. 18945223 10.1111/j.1530-0277.2008.00810.x PMC2643347

[2] R. W. Hingson , T. Heeren , and M. R. Winter , “Age at Drinking Onset and Alcohol Dependence: Age at Onset, Duration, and Severity,” Archives of Pediatrics & Adolescent Medicine 160, no. 7 (2006): 739–746. 16818840 10.1001/archpedi.160.7.739

[3] J. McCambridge , J. McAlaney , and R. Rowe , “Adult Consequences of Late Adolescent Alcohol Consumption: A Systematic Review of Cohort Studies,” PLoS Medicine 8, no. 2 (2011): e1000413. 21346802 10.1371/journal.pmed.1000413 PMC3035611

[4] International Alliance for Responsible Drinking (IARD) , Actions to Prevent Underage Drinking (IARD, 2023), https://cms.iard.org/getmedia/22ec2ef9‐f7bd‐41cf‐8896‐1a4f4f6ec015/04102023‐Actions‐to‐prevent‐underage‐drinking‐brochure.pdf.

[5] J. Inchley , D. Currie , S. Budisavljevic , et al., editors. Spotlight on Adolescent Health and Well‐Being. Findings From the 2017/2018 Health Behaviour in School‐Aged Children (HBSC) Survey in Europe and Canada, International Report: Key Findings, vol. 1 (WHO Regional Office for Europe, 2020).

[6] M. Fujii , Y. Kuwabara , A. Kinjo , et al., “Trends in the Co‐Use of Alcohol and Tobacco Among Japanese Adolescents: Periodical Nationwide Cross‐Sectional Surveys 1996‐2017,” BMJ Open 11, no. 8 (2021): e045063. 10.1136/bmjopen-2020-045063 PMC8340282 34348945

[7] Ministry of Health, Labour and Welfare , Health Japan 21 (the Third Term): Reference Materials (Ministry of Health, Labour and Welfare, 2023), https://www.mhlw.go.jp/content/001426890.pdf.

[8] A. Keski‐Rahkonen , J. Kaprio , A. Rissanen , M. Virkkunen , and R. J. Rose , “Breakfast Skipping and Health‐Compromising Behaviors in Adolescents and Adults,” European Journal of Clinical Nutrition 57, no. 7 (2003): 842–853. 12821884 10.1038/sj.ejcn.1601618

[9] J. Berro , M. Akel , S. Hallit , and S. Obeid , “Relationships Between Inappropriate Eating Habits and Problematic Alcohol Use, Cigarette and Waterpipe Dependence Among Male Adolescents in Lebanon,” BMC Public Health 21, no. 1 (2021): 140. 33446162 10.1186/s12889-021-10184-2 PMC7809860

[10] S. Pengpid , K. Peltzer , T. T. Nguyen‐Thi , and I. Jayasvasti , “Meal Skipping Among Adolescents in The Philippines: Prevalence, Associated Factors, and Associations With Dietary, Mental Health, and Health Risk Behavioural Outcomes,” Nutrition Journal 24, no. 1 (2025): 58. 40221786 10.1186/s12937-025-01118-4 PMC11992802

[11] B. P. Hasler and D. B. Clark , “Circadian Misalignment, Reward‐Related Brain Function, and Adolescent Alcohol Involvement,” Alcoholism, Clinical and Experimental Research 37, no. 4 (2013): 558–565. 23360461 10.1111/acer.12003 PMC3843484

[12] M. M. Wong , K. J. Brower , H. E. Fitzgerald , and R. A. Zucker , “Sleep Problems in Early Childhood and Early Onset of Alcohol and Other Drug Use in Adolescence,” Alcoholism, Clinical and Experimental Research 28, no. 4 (2004): 578–587. 15100609 10.1097/01.alc.0000121651.75952.39

[13] S. G. Nash , A. McQueen , and J. H. Bray , “Pathways to Adolescent Alcohol Use: Family Environment, Peer Influence, and Parental Expectations,” Journal of Adolescent Health 37, no. 1 (2005): 19–28. 10.1016/j.jadohealth.2004.06.004 15963903

[14] K. A. Bartel , M. Gradisar , and P. Williamson , “Protective and Risk Factors for Adolescent Sleep: A Meta‐Analytic Review,” Sleep Medicine Reviews 21 (2015): 72–85. 25444442 10.1016/j.smrv.2014.08.002

[15] Y. Otsuka , Y. Kaneita , A. P. Spira , et al., “Trends in Sleep Problems and Patterns Among Japanese Adolescents: 2004 to 2017,” Lancet Regional Health 9 (2021): 100107. 10.1016/j.lanwpc.2021.100107 PMC8315371 34327435

[16] S. J. Zhou , L. G. Zhang , L. L. Wang , et al., “Prevalence and Socio‐Demographic Correlates of Psychological Health Problems in Chinese Adolescents During the Outbreak of COVID‐19,” European Child & Adolescent Psychiatry 29, no. 6 (2020): 749–758. 32363492 10.1007/s00787-020-01541-4 PMC7196181

[17] G. Bennett , E. Young , I. Butler , and S. Coe , “The Impact of Lockdown During the COVID‐19 Outbreak on Dietary Habits in Various Population Groups: A Scoping Review,” Frontiers in Nutrition 8 (2021): 626432. 33748175 10.3389/fnut.2021.626432 PMC7969646

[18] H. Morioka , O. Itani , Y. Kaneita , et al., “Associations Between Sleep Disturbance and Alcohol Drinking: A Large‐Scale Epidemiological Study of Adolescents in Japan,” Alcohol 47, no. 8 (2013): 619–628. 24188738 10.1016/j.alcohol.2013.09.041

[19] Y. Osaki , T. Tanihata , T. Ohida , et al., “Decrease in the Prevalence of Adolescent Alcohol Use and Its Possible Causes in Japan: Periodical Nationwide Cross‐Sectional Surveys,” Alcoholism, Clinical and Experimental Research 33, no. 2 (2009): 247–254. 18986382 10.1111/j.1530-0277.2008.00822.x

[20] H. Morioka , O. Itani , Y. Osaki , et al., “The Association Between Alcohol Use and Problematic Internet Use: A Large‐Scale Nationwide Cross‐Sectional Study of Adolescents in Japan,” Journal of Epidemiology 27, no. 3 (2017): 107–111. 28142042 10.1016/j.je.2016.10.004 PMC5350616

[21] K. Yoshida , H. Kanda , T. Hisamatsu , et al., “Association and Dose‐Response Relationship Between Exposure to Alcohol Advertising Media and Current Drinking: A Nationwide Cross‐Sectional Study of Japanese Adolescents,” Environmental Health and Preventive Medicine 28 (2023): 58. 37766544 10.1265/ehpm.23-00127 PMC10569966

[22] A. Erol and V. M. Karpyak , “Sex and Gender‐Related Differences in Alcohol Use and Its Consequences: Contemporary Knowledge and Future Research Considerations,” Drug and Alcohol Dependence 156 (2015): 1–13. 26371405 10.1016/j.drugalcdep.2015.08.023

[23] M. A. Short and N. Weber , “Sleep Duration and Risk‐Taking in Adolescents: A Systematic Review and Meta‐Analysis,” Sleep Medicine Reviews 41 (2018): 185–196. 29934128 10.1016/j.smrv.2018.03.006

[24] M. Takakura , M. Miyagi , and A. Kyan , “Changes in the Prevalence of Health‐Risk Behaviors Among Japanese Adolescents Before and During the COVID‐19 Pandemic: 2002–2021,” School Health 19 (2023): 14–25, https://www.jstage.jst.go.jp/article/jash/19/0/19_SH_125/_article.

[25] H. Inaba , F. Hoshino , K. Takano , et al., “Impact of the Coronavirus Disease 2019 Pandemic on Leisure Screen Time and Eating Habits of Japanese High School Students: A Comparison Between Before and During the Pandemic,” Healthcare (Basel) 11, no. 9 (2023): 1265. 37174807 10.3390/healthcare11091265 PMC10177775

[26] N. Aono , A. Higashiyama , H. Suzuki , et al., “Associations Between Mental Health and Lifestyle Changes During the COVID‐19 Pandemic in a General Japanese Population: Nippon DATA2010,” Environmental Health and Preventive Medicine 30 (2025): 28. 40301095 10.1265/ehpm.24-00292 PMC12041440

[27] L. Wu and T. Shimizu , “Analysis of the Impact of Non‐Compulsory Measures on Human Mobility in Japan During the COVID‐19 Pandemic,” Cities 127 (2022): 103751. 35601133 10.1016/j.cities.2022.103751 PMC9114008

[28] E. Han , M. M. J. Tan , E. Turk , et al., “Lessons Learnt From Easing COVID‐19 Restrictions: An Analysis of Countries and Regions in Asia Pacific and Europe,” Lancet (London, England) 396, no. 10261 (2020): 1525–1534. 32979936 10.1016/S0140-6736(20)32007-9 PMC7515628

