# Supplementary material for: Lifestyle Factors and Current Alcohol Consumption Among Japanese Adolescents During the COVID‐19 Pandemic: A Nationwide Cross‐Sectional Study
Source: Neuropsychopharmacol Rep. 2026 Jan 11;46(1):e70089. doi: 10.1002/npr2.70089 (PMC12793038; doi:10.1002/npr2.70089)
Supplement: Supplementary file 1 — Data S1: npr270089‐sup‐0001‐Supinfo.docx. [file NPR2-46-e70089-s001.docx]

**Supplementary Materials**

**Variable Definitions, Coding Rules, and Analytic Workflow**

This supplementary file provides detailed descriptions of variable definitions, recoding rules, missing data handling, and the analytic workflow to enhance the reproducibility and transparency of this study.

**1. Outcome variable**

**Current alcohol consumption**

- Definition: Drinking alcohol on at least one day in the past 30 days.
- Coding rule: 1 = ≥1 day; 0 = 0 days.

**2. Demographic variables**

- Sex: Boy / Girl
- Grade: 7th–12th grade (categorical variable)
- School location:
  - Eastern Japan (Hokkaido, Tohoku, Kanto)
  - Central Japan (Hokuriku, Koshinetsu, Tokai)
  - Western Japan (Kansai, Chugoku, Shikoku, Kyushu, Okinawa)

**3. Lifestyle and psychosocial covariates**

Current smoking

- Definition: Tobacco use on at least one day in the past 30 days.
- Coding rule: 1 = Yes; 0 = No.

Bedtime

- Categories: Before midnight / After midnight.

Enjoyment of school life

- Response options: Yes / Neutral / No.
- Treatment in analysis: Included as a categorical variable with three levels.

Parental alcohol use

- Question:
  “Have you ever felt uncomfortable when your parent(s) drank alcohol?”
- Coding rule:
  Responses “Yes” or “No” were classified as having drinking parents;
  the response “My parent(s) do not drink” was classified as having nondrinking parents.

Depressed mood (past 30 days)

- Response options: Never / Rarely / Sometimes / Often.
- Coding rule:
  Never or Rarely = 0 (No);
  Sometimes or Often = 1 (Yes).

**4. COVID-19–Related Lifestyle Changes (Main Explanatory Variables)**

Participants responded to the following multiple-response question:
“Due to the COVID-19 pandemic in 2020, including school closures and other unusual circumstances, did you experience any negative effects? Please check all that apply.”

Each response option was treated as an independent binary variable (checked = 1, not checked = 0), and respondents were allowed to select multiple options.

For the present analysis, the following seven items were selected and treated as independent binary variables:

- Irregular sleep patterns
- Irregular dietary habits
- Difficulty in study
- Increased screen time
- Accumulated stress
- Physical inactivity
- None of the above

The option “None of the above” was not included as an explanatory variable in the regression analyses.

Operational definitions

- Irregular sleep patterns: A self-reported subjective change in sleep rhythm, including shorter or longer sleep duration and reversal of the sleep–wake cycle (day–night reversal).
- Irregular dietary habits: Disruptions in eating patterns, including changes in meal frequency, timing, or nutritional balance.
- Increased screen time: Increased time spent on gaming or internet use; educational screen use (e.g., online classes) was excluded.

**5. Handling of Missing Data**

Participants with missing data on key variables (current alcohol consumption, sex, grade, or COVID-19–related lifestyle items) were excluded from the analytic dataset. For the multivariable logistic regression analyses, a complete-case approach was applied.

The final analytic sample comprised 15,351 participants after exclusion of 198 participants (1.3% of the total sample) who had missing values in one or more covariates. The numbers of missing values were as follows:

- Bedtime: n = 9
- Enjoyment of school life: n = 61
- Current smoking: n = 127
- Parental alcohol use: n = 59
- Depressed mood: n = 24

Because some participants had more than one missing value, the total number of excluded participants was less than the sum of missing values. Given the small proportion of missing data, the impact on the study findings was considered negligible.

**6. Statistical Analysis Workflow**

All analyses were conducted using Stata/SE version 18.5 (StataCorp LLC, College Station, TX, USA) and accounted for the one-stage stratified cluster sampling design, with school as the primary sampling unit and the application of survey sampling weights.

The analytic workflow was as follows:

1. Selection of the analytic dataset after applying exclusion criteria based on missing key variables.
2. Calculation of survey-weighted descriptive statistics for all study variables.
3. Examination of bivariate associations between current alcohol consumption and each demographic or lifestyle factor using chi-square tests.
4. Fitting of survey-weighted multivariable logistic regression models to estimate odds ratios (ORs) and 95% confidence intervals (CIs) for the associations between COVID-19–related lifestyle factors and current alcohol consumption.
5. Conducting sex-stratified analyses to examine potential sex differences.
6. Inclusion of interaction terms (sex × lifestyle factor) to formally test effect modification by sex.
7. Performance of model diagnostics using the Hosmer–Lemeshow goodness-of-fit test, and assessment of multicollinearity using variance inflation factors (VIFs).

A two-tailed *p* value < 0.05 was considered statistically significant. Odds ratios and 95% confidence intervals were rounded to two decimal places. Because all lifestyle variables were selected a priori based on conceptual relevance, no formal adjustment for multiple testing was applied.

**7. Supplementary Analyses**

Table S1. Sensitivity analysis: Associations between lifestyle factors and current alcohol consumption additionally adjusted for bedtime and depressed mood.

|  | All (n=15,320) | Boys (n=7,524) | Girls (n=7,796) |  |
| --- | --- | --- | --- | --- |
|  | OR (95% CI) | OR (95% CI) | OR (95% CI) | *p* for interaction |
| Irregular sleep patterns | 1.40 (1.06-1.83) * | 1.15 (0.76-1.74) | 1.70 (1.27-2.29) ** | 0.012 |
| Irregular dietary habits | 1.60 (1.14-2.25) ** | 1.42 (0.84-2.39) | 1.69 (1.07-2.68) * | 0.380 |
| Difficulty in study | 1.00 (0.70-1.43) | 0.90 (0.50-1.63) | 1.12 (0.72-1.73) | 0.366 |
| Increased screen time | 1.25 (0.96-1.62) | 1.43 (0.98-2.08) | 1.08 (0.79-1.48) | 0.275 |
| Accumulated stress | 0.88 (0.66-1.16) | 0.70 (0.41-1.18) | 1.11 (0.74-1.68) | 0.140 |
| Physical inactivity | 1.09 (0.84-1.43) | 1.07 (0.76-1.51) | 1.11 (0.75-1.63) | 0.723 |

Models were additionally adjusted for bedtime (before/after midnight) and depressed mood (past 30 days), which were excluded from the primary models because of conceptual overlap with the main lifestyle measures. Because participants with missing responses for bedtime or depressed mood were excluded, the sample size for this sensitivity analysis was n=15,320.

Current alcohol consumption was defined as drinking alcohol on at least one day in the past 30 days.

OR = odds ratio; CI = confidence interval.

* *p* < 0.05; ** *p* < 0.01

Table S2. Associations between lifestyle factors and current alcohol consumption stratified by school level (junior high school and senior high school).

|  | Junior high school (n = 7,975) | Senior high school (n = 7,376) |  |
| --- | --- | --- | --- |
|  | OR (95% CI) | OR (95% CI) | *p* for interaction |
| Irregular sleep patterns | 1.33 (0.81-2.19) | 1.59 (1.15-2.19) ** | 0.396 |
| Irregular dietary habits | 1.04 (0.62-1.76) | 2.04 (1.33-3.14) ** | 0.027 |
| Difficulty in study | 1.01 (0.62-1.66) | 1.06 (0.61-1.84) | 0.859 |
| Increased screen time | 1.21 (0.83-1.76) | 1.34 (0.92-1.96) | 0.736 |
| Accumulated stress | 1.02 (0.67-1.54) | 0.88 (0.55-1.40) | 0.654 |
| Physical inactivity | 1.13 (0.77-1.64) | 1.12 (0.77-1.64) | 0.910 |

Models were adjusted for sex, grade level, school location, enjoyment of school life, current smoking, and parental alcohol use.

Current alcohol consumption was defined as drinking alcohol on at least one day in the past 30 days.

OR = odds ratio; CI = confidence interval.

* *p* < 0.05; ** *p* < 0.01

Table S3. Associations between lifestyle factors and current alcohol consumption stratified by survey mode (paper-based and web-based surveys).

|  | Paper-based (n = 9,064) | Web-based (n = 6,287) |  |
| --- | --- | --- | --- |
|  | OR (95% CI) | OR (95% CI) | *p* for interaction |
| Irregular sleep patterns | 1.30 (0.95-1.79) | 2.03 (1.37-3.02) ** | 0.088 |
| Irregular dietary habits | 1.38 (0.91-2.07) | 2.40 (1.36-4.23) ** | 0.154 |
| Difficulty in study | 0.75 (0.48-1.16) | 1.84 (1.07-3.16) * | 0.011 |
| Increased screen time | 1.09 (0.82-1.44) | 1.94 (1.31-2.87) ** | 0.015 |
| Accumulated stress | 0.80 (0.60-1.06) | 1.18 (0.55-2.53) | 0.318 |
| Physical inactivity | 1.20 (0.85-1.70) | 0.99 (0.61-1.63) | 0.392 |

Models were adjusted for sex, grade level, school location, enjoyment of school life, current smoking, and parental alcohol use.

Current alcohol consumption was defined as drinking alcohol on at least one day in the past 30 days.

OR = odds ratio; CI = confidence interval.

* *p* < 0.05; ** *p* < 0.01
